# Standard methods for estimating mechanical work in high-intensity functional training: an exercise compendium

**DOI:** 10.3389/fspor.2026.1974999

**Published:** 2026-10-27

**Authors:** Gerald T. Mangine, Caralynn Doese, Adam R. Jajtner

**Affiliations:** 1 Exercise Science and Sport Management, Kennesaw State University, Kennesaw, GA, United States; 2 Research Center on Human Sport Performance and Wellbeing, Kennesaw State University, Kennesaw, GA, United States; 3 Exercise Physiology, Kent State University, Kent, OH, United States

**Keywords:** anthropometrics, crossFit®, movement standards, physiological stress, programming strategies, work rate

## Abstract

High-intensity functional training (HIFT) presents unique challenges for quantifying training load due to the variability of exercises, movement standards, and workout structures. Traditional workload metrics used in strength and endurance training are difficult to apply because HIFT workouts rarely repeat, combine multiple modalities, and lack consistent recommendations for sequencing or volume. This paper provides standardized methods for estimating the mechanical work of common HIFT exercises using simplified biomechanical models, published movement standards, and average anthropometric data. Equations are presented for weightlifting, plyometric, calisthenic, gymnastics, and monostructural modalities, along with examples demonstrating how to calculate total work and work rate in representative workouts. Although metabolic work cannot yet be reliably estimated during HIFT, standardized estimates of positive external mechanical work provide a reproducible framework for comparing workout prescriptions, organizing program design, and reporting mechanical demands. These methods are not substitutes for direct kinetic or kinematic measurement and have not yet been validated against criterion measurements of mechanical work.

## Introduction

The “sport of fitness” describes one of the competitive aspects of high-intensity functional training (HIFT). It refers to personal growth in fitness and a sport that seeks to find the fittest athletes. Indeed, the title given to winners of the most popular HIFT competition, the CrossFit® Games^TM^, is “The Fittest on Earth”. Individuals and teams earn this title by participating in a multistage, international competition that has them complete several unique workouts purportedly designed to test one or more aspects of fitness (1, 2). Each workout may include a different number and combination of a wide variety of exercise modalities, and each exercise can be prescribed a different load (if applicable) and number of repetitions. Specific workouts are rarely repeated (3), which leaves the instruction to complete assigned work as fast as possible as the only common characteristic. During the earlier stages of competition, competitors have the liberty to complete workouts at their normal training facility and on their own time within competition parameters, whereas later stages are in-person events that program several workouts within and across consecutive days. The latter is also a common format among third-party competitions (e.g., Rogue Invitational, Wodapalooza, The Granite Games), whereas regular, non-competition HIFT sessions are limited to a single workout-of-the day (WOD).

Regardless of whether a WOD is designed for competition or training, few recommendations have been offered for how consecutive workouts should be organized. The original philosophy recommended a balanced rotation in the number and combination of modalities programmed within a training week (2) but said nothing about total training hours or workloads. It was simply acknowledged that fatigue would accumulate across sessions, and that time off would be needed for recovery. In more traditional training styles, strength and conditioning professionals vary and sequence training workloads within and across mesocycles to overload targeted physiology or facilitate recovery (4, 5). Coaches adjust workloads by adding or subtracting training sessions, exercises and modalities, and by altering exercise order, intensity and volume combinations, and rest intervals. The process is straightforward. Because specific workouts appear frequently (4, 6, 7), coaches only need to make minor adjustments after establishing the initial workloads. It is not as simple with HIFT because few surface-level programming variables are the same between WODs and this makes minor adjustments to workload difficult (or impossible) to notice.

There is cause for concern when adjustments to programming are unclear. A recent scoping review of 60 studies reporting acute responses to 35 different HIFT workouts noted inconsistent research methods and extreme variability in physiological responses (8). Sample populations and outcome variables were often different and poorly described, and few conclusions could be made about expected physiological responses. One was that intensity, as measured by heart rate and lactate responses, was consistent with the terms “high intensity” or “vigorous” effort (9). Though technically correct, the qualifying ranges for these terms are large and cannot distinguish the true difficulty between WODs. Coaches and athletes cannot program with purpose if they cannot associate a specific response with a precise intensity or workload. It becomes impossible to predict whether the intended stimulus is being met or exceeded, or whether training is sufficiently balanced. When training programs do not (or cannot) consider the simultaneous physiological effects of a training stimulus, there is an elevated risk of overtraining, maladaptation, and/or injury (10).

This concern is not new. Much attention has been given to HIFT injury (11–14), and the evidence does not suggest an enhanced risk compared to more traditional training styles and sports. The risk seems most affected by a lack of training experience, training frequency, and/or coaching quality. Unfortunately, strategies employed by elite coaches remain unavailable. Realistic performance and/or capacity targets are known through normative scores (15–17), but none of these have been conclusively tied to any physiological responses. Given the variety of potential WODs to be studied, examining responses to each is not realistic. A more reasonable approach would be to classify workouts based on intensity, workload, and complexity, and then examine for consistent responses amongst workout classifications. The findings would empower coaches with a means of organizing workout designs based on classification and expected response, along with the ability to make small but noticeable adjustments to fit the needs of their athletes and periodization strategy. Anecdotal evidence suggests that coaches attempt to do this by feel, but not through objective calculations (18, 19). Though we strongly encourage using observation and personal experience to help guide training, these should be paired with objective data to reduce the margin for error and produce a more efficient programming strategy.

### Understanding total workload

Mechanical and metabolic work are key components of the total amount of work completed during exercise (20–23). Mechanical work is the product of force and displacement in the direction of that force and is conventionally expressed in joules (J; N·m). Because the simplified equations presented in this compendium generally use load mass (kg) multiplied by vertical displacement (m), results are reported as kilogram-force meters (kgf·m), where 1 kgf·m = 9.80665 J. Thus, when a mass is raised vertically through a given distance, mass × displacement provides the numerical estimate in kgf·m, whereas mass × gravitational acceleration×displacement provides the equivalent value in joules. The estimates presented here characterize positive external mechanical work and should not be interpreted as direct measures of force, internal joint work, tissue stress or strain, or musculoskeletal loading.

Conversely, metabolic work reflects the production and expenditure of the adenosine triphosphate (ATP), which is necessary to perform mechanical work within a given duration (24). This value reflects the physiological cost of performing mechanical work and knowing it provides an approximation of the amount of fatigue induced by exercise. It is the necessary counterpart to fully appreciate exercise-induced musculoskeletal damage. Ideally, programming is accurately and purposefully modified when both the expected and/or completed mechanical and metabolic work are known but this is unlikely.

Direct measurement or instrument-based derivation of mechanical work generally requires technologies such as force plates, three-dimensional high-speed cameras, or linear position transducers to quantify force and/or displacement during each effort (25–27). Collected values can then be transferred to a modifiable spreadsheet for aggregation, analysis, and program modification. Though simple enough, the financial costs, knowledge, and skill required to own and operate relevant technologies may deter some from ever incorporating this step into programming. Moreover, the variety of modalities that might appear in HIFT would often require multiple technologies to assess force expression in each movement assigned to a workout. Still, some deterrents can be bypassed by pairing the principles of physics with readily available information to estimate mechanical work. For many exercises that appear in HIFT programming, positive external mechanical work is dominated by vertical displacement against the force of gravity (9.8 m·sec^−2^) (21, 23). The expected displacement for these exercises can be approximated because their correct execution depends on adherence to published movement standards (3). Actual displacement will vary across individuals, but it can generally be estimated based on the exercise's starting and end points for both the person and the object moved. When expressed in kgf·m, positive external mechanical work is approximated as the sum of each load multiplied by its respective displacement (21, 23).

The caveat to this approximation is that it assumes the exercise's motion occurs in a linear direction, which is not the case for movements that commonly deviate from a linear trajectory (i.e., during Olympic lifts and kettlebell exercises) (28, 29). Exact displacement, velocity, and acceleration can be verified and corrected through video analysis software (16, 17, 30, 31), though this extremely tedious solution may not be worthwhile in sub-elite competitors. Precision may not even be necessary if consistent methods are employed.

Accordingly, the equations presented in this compendium should be interpreted as standardized estimates of mechanical work rather than direct measurements. The equations have not been validated against criterion measurements derived from force platforms, three-dimensional motion capture, or comparable methods. Their purpose is to provide a reproducible field-based estimate when direct measurement is impractical. Accuracy will depend on the anthropometric, movement, and technical assumptions used in each calculation.

Without standardized physical parameters (e.g., lowering a barbell to the safety bars within a squat rack), displacement variations across repetitions are inevitable. Trainees might randomly or intentionally alter their range of motion due to preference, skill, or physical limitations, and it can become unclear whether such deviations lead to over- or underestimation, as compensatory movements may offset or exaggerate the range. While all are potential sources of error, that error can be assumed to be consistent if the assessment is limited to a single person. Thus, comparisons between individuals may be unreliable, whereas repeated comparisons within the same person are more likely to be consistent.

Metabolic work is more difficult to measure and estimating it may always be problematic. The gold standard for quantifying metabolic costs requires exercise to be performed within a chamber that can measure heat production (32, 33), a logistically difficult endeavor for many researchers and coaches. Indirect calorimetry is more common, but also presents logistical challenges, especially during HIFT. The method utilizes a metabolic cart that measures the ratio of exhaled carbon dioxide to inhaled oxygen to estimate caloric expenditure and the most likely source of ATP (i.e., from glucose and/or triglycerides) (33). This method lacks utility in a HIFT setting because even the less reliable, portable methods require the trainee to wear a mask that physically impairs movement. Consequently, few studies have attempted indirect calorimetry during a HIFT-style workout, and instead, have settled on post-exercise quantification (8). Without a true value, the validity of any estimate of the metabolic costs during HIFT will suffer.

It may be possible to infer metabolic costs with certain information about the person and workout and then use this information as a placeholder until it can be validated against “true” values. Several factors contribute to the metabolic stress of exercise (24, 34) and many of these are not mutually exclusive:

- Individual training age and status, fitness and health status
- Nutrition and hydration status
- Relative intensity of the mechanical stress
- Cumulative total of the duration and frequency of individual efforts
- Programming and environmental context
- Psychological motivation, as well as voluntary and perceived effort

Much research is needed before the influence of many of these factors on HIFT performance is well understood. Likewise, standard methods for inferring metabolic costs are not established, but these could happen much sooner. Assuming consistent effort, motivation, and “normal” environmental, nutritional/hydration, and health conditions, a coach might reasonably estimate the metabolic costs of a HIFT workout from prescribed mechanical work, relative intensity, and expected duration. Aside from the methods for calculating mechanical work presented here, standard methods for estimating relative intensity and expected duration are still in development.

It was recently proposed that the relative intensity of HIFT-style workouts should be likened to how pace is used in endurance athletes (35). Traditionally, relative intensity for weightlifting exercises is defined as the programmed load's relationship to a fixed standard (i.e., 1-RM, body mass) (7). Calisthenics might also use a fixed standard like body mass, or they could also be related to a repetition-maximum (e.g., complete 50% of your repetition-maximum in pull-ups), and physiological characteristics such as aerobic capacity and lactate thresholds are often cited as the standards for describing relative intensity in endurance modalities (6, 36). However, if the vast number of exercises that would require assessment was not sufficiently deterrent, the logistical complications of monitoring these during a WOD makes all these methods impractical for HIFT athletes. Instead, HIFT athletes might shift their definition of intensity to match a common practice amongst endurance athletes by describing effort as a function of racing pace. For example, training at a pace that is above, below, or at their racing pace for a given duration/distance to facilitate one or more specific adaptations (6). This strategy appears suitable for HIFT because most workouts are formatted to encourage trainees to complete assigned work as quickly as possible (3, 35). With these concepts in mind, the average repetition completion rate in a workout, relative to the individual's ability to perform assigned exercises at maximal speed, could universally describe the intensity of any HIFT workout. Validation data has been presented on this topic (37) but how maximal speed should be defined remains a work in progress. Remaining questions ask if the definition should vary across exercises, and if the approach should consider context (e.g., maximal speed of an entire round, not the individual exercises).

In addition to determining relative intensity, a related application of the repetition completion rate concept might help estimate workout duration, and thus, the rate of completing assigned work. By knowing the average amount of time required to complete individual repetitions of each exercise, coaches could forecast time-to-completion when the total workload is fixed, or the number of repetitions completed when a circuit is meant to be completed for “*as many repetitions as possible*” (AMRAP) within a fixed duration. This combination, paired with an independently determined measure of relative intensity, could be investigated as one component of a broader characterization of workout demands. However, it should not be assumed to directly represent physiological stress or fatigue, and any such estimate would likely require adjustment for training status and workout design. For example, the number of assigned repetitions and/or load magnitude would likely impact an exercise's average repetition completion rate (38), and even with careful planning and strict adherence, the cumulative effects of minor disturbances and variation in tempo would limit precision. Nevertheless, workload completion rate would provide a universally relevant and comprehensive value for equating HIFT-style workouts and better understanding observed responses. However, too much about this hypothesis remains unexplored for worthwhile estimates to be presented here.

## Standardized methods for estimating mechanical work

It is important to acknowledge that the idea of quantifying HIFT workloads is not new. While supporting his definition of fitness in the Level 1 Training Guide (2), Glassman provides an example for calculating the work and power expressed by a 1.82 m, 90 kg male performing the benchmark WOD known as “Fran” (21, 15, and 9 repetitions of barbell thrusters and pull-ups). He intended on giving coaches a method for objectively describing what it meant to perform the workout and improve one's score. However, the example oversimplified the work calculation, incorrectly applied the term “power”, and provided no additional guidance for more complicated exercises and workouts. The lack of further guidance may be the most important reason why coaches do not calculate HIFT workloads often. As we have described, an accurate estimate or account of workload must carefully consider all factors related to the biomechanical and metabolic stress of each exercise within a WOD. Although these concepts are covered by most training certifications (39), advanced knowledge is not a prerequisite to be a coach. Not all coaches will feel comfortable in their understanding and ability to accurately estimate work completed on a given exercise. That, or the sheer volume of potential exercises to be estimated may be viewed as a tedious and daunting task, especially if one does not fully appreciate the value in performing this task. Therefore, the purpose of this section is to eliminate the most difficult steps in this process and provide coaches with a simplified resource that will aid in the estimation of the mechanical work required of single repetitions in the most common exercises programmed in HIFT. Coaches may use this tool when writing generalized programming for several people, or the provided calculations and explanations can be used to provide more specific estimates when programming for individual athletes.

The following subsections will provide detailed descriptions for estimating total concentric displacement (m) and force expression (kg) for every exercise that has been programmed into each CrossFit® Open workout between 2011 and 2025 (3, 40). The estimate for concentric displacement will be based on the current movement standards described in CrossFit® Open Workout cards and enforced by certified judges. Although we acknowledge that the standards described for some exercise allow for some leeway in execution, and thus, completed mechanical work, our estimates will be made to quantify what is minimally correct (i.e., exactly parallel for a parallel squat). The concentric distance covered on each exercise is supported by an explanation of the calculation using standard anthropometric information (i.e., stature, body mass) about the average sized male (body mass = 85.6 kg, height = 177.2 cm) and female (body mass = 62.6 kg, height = 163.6 cm) athlete appearing in competition (15–17, 41). Regions of interest lengths of the head, neck, torso, upper- and lower-arm, wrist/hand, upper- and lower-leg, and ankle/foot were estimated for each model using previously described techniques (42–46), and these are illustrated in Figures 1A,B. These average anthropometrics were used to estimate concentric displacement for common exercises, and then displacement was considered alongside the mass of the associated external load (if applicable) and relevant regions of interest of the person, determined as percentages of total mass according to (47), to find work (kgf·m).

**Figure 1:**
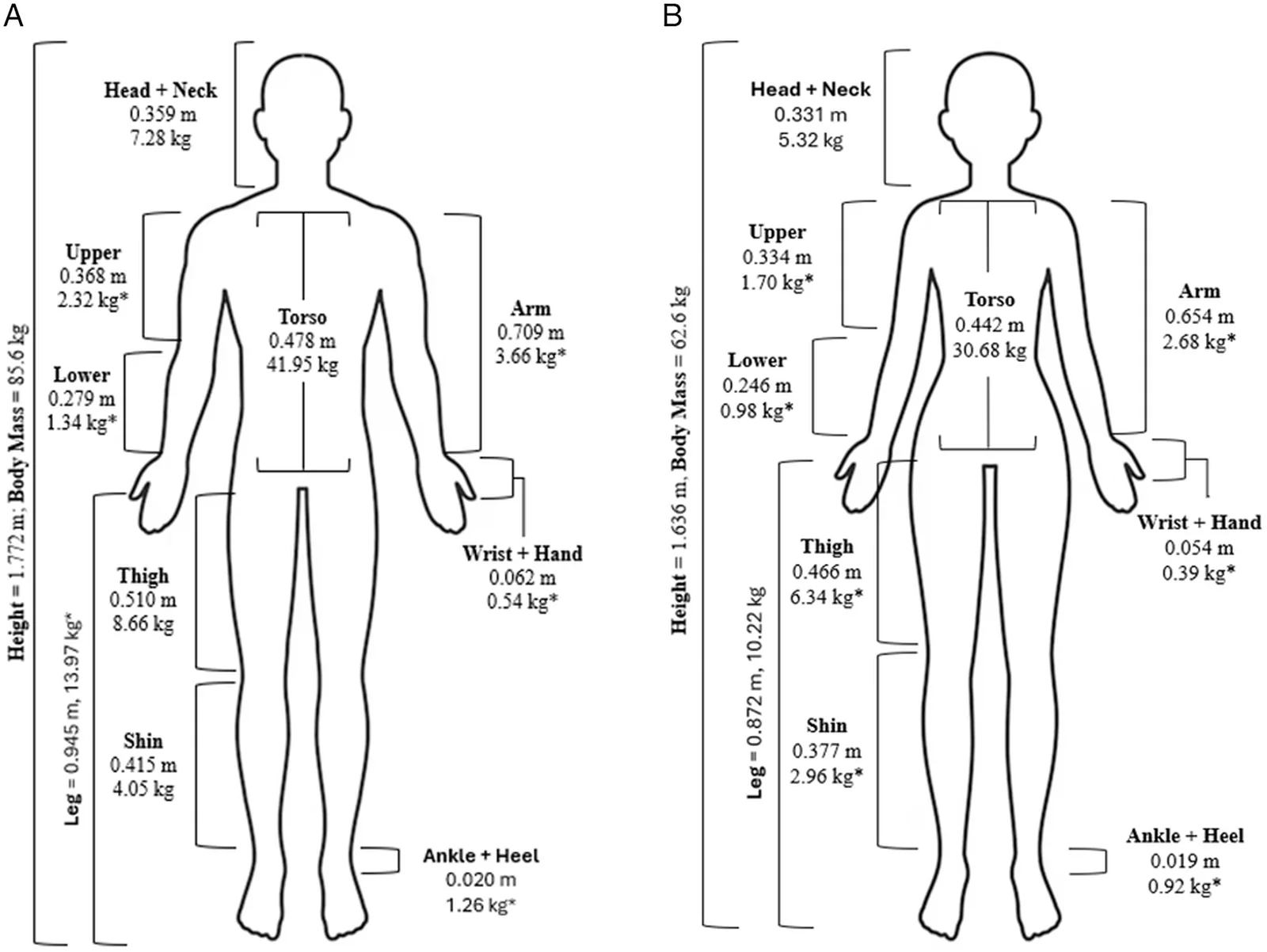
Regional estimates of height and mass for **(A)** men and **(B)** women. * = mass of individual limbs.

Model inputs were grouped into three categories based on how they were established. First, directly supported parameters were based on published research, manufacturer specifications, or published competition movement standards (e.g., body-segment proportions, prescribed box or target heights, and ergometer constants). Second, derived parameters were calculated from these sources using geometric or biomechanical relationships (e.g., regional lengths, squat displacement, and leg radius). Third, when published data were unavailable or movement standards allowed for multiple valid techniques, standardization assumptions were made by the authors (e.g., minimum range of motion, assumed countermovement depth, minimum jump clearance, and conservation of elastic energy). These assumptions are intended to standardize estimates, not represent measured values. Observed values should replace these assumptions when they are available.

A sensitivity analysis was also performed to determine how anthropometric variation affected standardized mechanical-work estimates. Five commonly programmed exercises were selected to represent each major classification: barbell thrusters (weightlifting), box jumps (plyometrics), burpees (calisthenics), kipping pull-ups (gymnastics), and rowing (continuous modality). For each sex, calculations were performed using the standard anthropometric model and six additional conditions: stature −1 SD, stature +1 SD, body mass −1 SD, body mass +1 SD, stature and body mass −1 SD, and stature and body mass +1 SD. Standard deviations reported by (41) were applied to the standard male and female anthropometric values used throughout this compendium. Stature-dependent body-segment lengths and displacements were scaled proportionally to the altered stature, whereas body-mass-dependent terms were recalculated while retaining the same segment-mass proportions used in the standard models. All other exercise-specific inputs and assumptions were held constant, including external loads, equipment dimensions, movement technique, and calculation coefficients. The analysis used 95-lb (43.1-kg) and 65-lb (29.5-kg) barbell thrusters, 24-in (0.610-m) and 20-in (0.508-m) box jumps, traditional burpees, traditional kipping pull-ups, and 55-calorie rowing for men and women, respectively. Sensitivity was expressed as the percentage difference in estimated work from the standard anthropometric model. Work rate was not reported separately because, when exercise duration is held constant, its proportional change is identical to that observed for mechanical work.

The following subsections provide more specific details about considerations made for each exercise and are organized based on the following classifications:

- Weightlifting (Any exercise that requires the athlete to move a barbell, dumbbell, or kettlebell)
- Plyometrics (Any exercise that involves jumping or projecting an object)
- Calisthenics (Any exercise where a percentage of body mass is the sole contributor to load, is performed on the floor requiring low-to-moderate skill)
- Gymnastics (Any exercise where a percentage of body mass is the sole contributor to load, is performed on the floor but requires a high amount of skill, or it is performed on pull-up bar, gymnastics rings, or a rope and requires moderate-to-high skill)
- Continuous modalities (rowing, cycling, skiing, running)

### Weightlifting exercises

Table 1 presents mechanical work equations for weightlifting exercises that have appeared in official HIFT competition programming (3), plus common variations that are likely to appear in training. These exercises most often utilize a barbell or dumbbell, and unless otherwise stated, the initial repetition always begins from the floor. For exercises that begin and must always return to the floor (e.g., deadlifts, power cleans, etc.), a distinction is made to account for greater concentric displacement caused by a lower starting position with a dumbbell (Women = 0.067 m; Men = 0.070 m) vs. a barbell (0.225 m). Starting points are set as the head radius of 35-lb and 50-lb rubber hex dumbbells or the radius of Olympic bumper plates (Rogue Fitness, Columbus, OH, USA). Displacement for the first repetition of exercises that do not return to the floor after the first repetition (e.g., hang power cleans, thrusters, jerks) are not described. Rather, the equations describe calculation procedures for all subsequent repetitions and notate recommended calculations for the first repetition. For example, a standard countermovement depth of 0.127 m (∼5 inches) was applied to all exercises from the hang position, and an initial deadlift calculation is recommended for these exercises due to the full-stop at the hang position requirement prior to initiating the first repetition (3). Because the number of times an athlete might drop the barbell within a set (i.e., to rest) cannot be predicted, all calculations assume unbroken sets. All standard displacements assume faithful execution of the minimum requirements of each exercise's established movement standard (3). For example, though athletes might squat well below parallel when performing squat cleans, the minimum standard requires them to squat to a parallel position. Coaches should be advised that the values presented here might underestimate workloads for trainees and athletes whose efforts cover a greater ROM than is required. Conversely, these values will overestimate workloads whenever ROM is shortened. That is, assuming shortened repetitions are validated when they would otherwise be considered “no reps” in competition (40).

**Table 1:** Mechanical work equations for common weightlifting exercises.

Exercise	Standard Body Mass	Standardized Concentric Displacement Estimate			Mechanical work formula	Notes
		Measurement	Men	Women		
Clean						
Power (Barbell)	88% Men = 75.3 kg Women = 55.1 kg	Height—Plate Radius—Head + Neck—(0.5 × Wrist + Hand)	1.157 m	1.053 m	(Body Mass × Quarter Squat Displacement) + (Barbell Mass × Standard Displacement)	Add one additional barbell deadlift to the first repetition of each set
from hang position		Arm + 0.127 m	0.836 m	0.781 m		
Power (Dumbbell)		Height—Dumbbell Radius—Head + Neck—(0.5 × Wrist + Hand)	1.315 m	1.205 m	(Body Mass × Quarter Squat Displacement) + (Dumbbell Mass × Standard Displacement)	Add one additional dumbbell deadlift to the first repetition of each set
from hang position		Arm + 0.127 m	0.836 m	0.781 m		
Squat (Barbell)		Height—Plate Radius – Head + Neck—(0.5 × Wrist + Hand)	1.157 m	1.053 m	(Body Mass × Parallel Squat Displacement) + (Barbell Mass × Standard Displacement)	Add one additional barbell deadlift to the first repetition of each set
from hang position		Arm + 0.127 m	0.836 m	0.781 m		
Deadlift						
Barbell	0%	(Leg + Torso)—Arm—(0.5 × Wrist + Hand)—Plate Radius	0.458 m	0.408 m	Barbell Mass × Standard Displacement	
Dumbbell		(Leg + Torso)—Arm—(0.5 × Wrist + Hand)—Dumbbell Radius	0.616 m	0.563 m	Dumbbell Mass × Standard Displacement	
Jerk	88% Men = 75.3 kg Women = 55.1 kg	Arm + Quarter Squat	0.858 m	0.790 m	(Body Mass × Quarter Squat Displacement) + (Barbell Mass × Standard Displacement)	Add one additional power clean or clean to the first repetition of each set
Push Press						
Shoulder-to-Overhead						
Shoulder Press	0%	Arm Length	0.709 m	0.654 m	Barbell or Dumbbell Mass × Standard Displacement	
Snatch						
Power (Barbell)	88% Men = 75.3 kg Women = 55.1 kg	(Height—Plate Radius—Head + Neck)—(0.5 × Wrist + Hand) + Torso	1.866 m	1.707 m	(Body Mass × Quarter Squat Displacement) + (Barbell Mass × Standard Displacement)	Add one additional barbell deadlift to the first repetition of each set
from hang position		(Torso × 2) + 0.127 m	1.083 m	1.011 m		
Power (Dumbbell)		(Height—Dumbbell Radius—Head + Neck—(0.5 × Wrist + Hand) + Arm	2.024 m	1.862 m	(Body Mass × Quarter Squat Displacement) + (Dumbbell Mass × Standard Displacement)	Add one additional dumbbell deadlift to the first repetition of each set
from hang position		(Torso × 2) + 0.127 m	1.083 m	1.011 m		
Squat (Barbell)		(Height—Plate Radius—Head + Neck)—(0.5*Wrist + Hand) + Torso	1.635 m	1.495 m	(Body Mass × Parallel Squat Displacement) + (Barbell Mass × Standard Displacement)	Add one additional barbell deadlift to the first repetition of each set
From hang position		(Torso × 2) + 0.127 m	1.083 m	1.011 m		
Squat						
Front rack	88% Men = 75.3 kg Women = 55.1 kg	Parallel Squat	0.591 m	0.539 m	(Body Mass × Standard Displacement) + (Barbell [or Dumbbell] Mass × Standard Displacement)	Add one power clean or clean repetition to each set to account for the barbell (or dumbbell) beginning from the floor.
Overhead						Add one power clean & jerk, snatch, or ground-to-overhead repetition to each set to account for the barbell beginning from the floor.
Thrusters	88% Men = 75.3 kg Women = 55.1 kg	Parallel Squat + Arm	1.300 m	1.193 m	(Body Mass × Parallel Squat Displacement) + (Dumbbell or Barbell Mass × Standard Displacement)	Add one power clean or clean repetition to each set to account for the barbell beginning from the floor.
Walking Lunge						
Overhead	84% Men = 71.9 kg Women = 52.6 kg	Shin + Ankle + Heel	0.435 m	0.396 m	[(Body Mass × Standard Displacement) + (Dumbbell Mass × Standard Displacement)] × (Distance ÷ Leg)	Add one power snatch repetition to each set to account for grip width and the barbell beginning from the floor.
Front rack						Add one power clean, power clean & jerk, snatch, or ground-to-overhead repetition to each set to account for the barbell beginning from the floor.

The displacement and value of both the external load and person (defined as a percentage of body mass) are both considered when calculating workload. Total vertical displacement summarizes what is quantified by the portion of each equation that accounts for the external load's contribution to mechanical work. The caveat to this approach is that it assumes the exercise's motion occurs in a linear direction, which is not the case for movements that commonly deviate from a linear trajectory (i.e., during Olympic lifts and kettlebell exercises) (28, 29). However, deviations along the sagittal plane are likely to have a greater impact on metabolic cost more distinctly than mechanical work (48), and these are impossible to predict from a generalized perspective. The total vertical displacement of the external load is multiplied by the assigned mass of the external load, and this is added to the vertical displacement of the athlete's center of mass (COM) multiplied by the relevant percentage of total body mass to find total mechanical workload for each repetition.

The determination threshold set for identifying the relevant percentage of body mass was based on squat depth. The only exceptions to when body mass was not considered a part of the mechanical work calculation were deadlift and shoulder press. This was because both exercises represent conditions where changes in the position of COM were almost entirely the consequence of changes to the external load's position. For all other exercises, standard displacements were calculated for three squat depths: Quarter Squat, Parallel Squat, and Deep Squat. For the parallel squats, previously identified average angles at the ankle, knee and hip during the front squat (49) were used to calculate the vertical distance from the floor to the knee, the knee to the hip and the hip to the shoulder. These distances were summed and then subtracted from the standing distance of the floor to the shoulder to obtain total displacement (Figure 2A). A similar approach was used to quantify the displacement of the Quarter Squat, except for the 45° angle being assigned to the knee and hip (Figure 2B). Deep squat displacement was calculated in a similar fashion, though, the angles used at the ankle, knee and hip were obtained from kinematic analysis (Kinovea; France) of a pistol squat (50) (Figure 2C). These squat depth thresholds were chosen because of the frequency with which the squatting motion occurs in common HIFT exercises (3) and their ability to distinguish minimum movement standards (e.g., the parallel requirement of a squat clean vs. the countermovement performed during a push press). Figure 2 illustrates the calculations of average displacement of all three squat depths for men and women.

**Figure 2:**
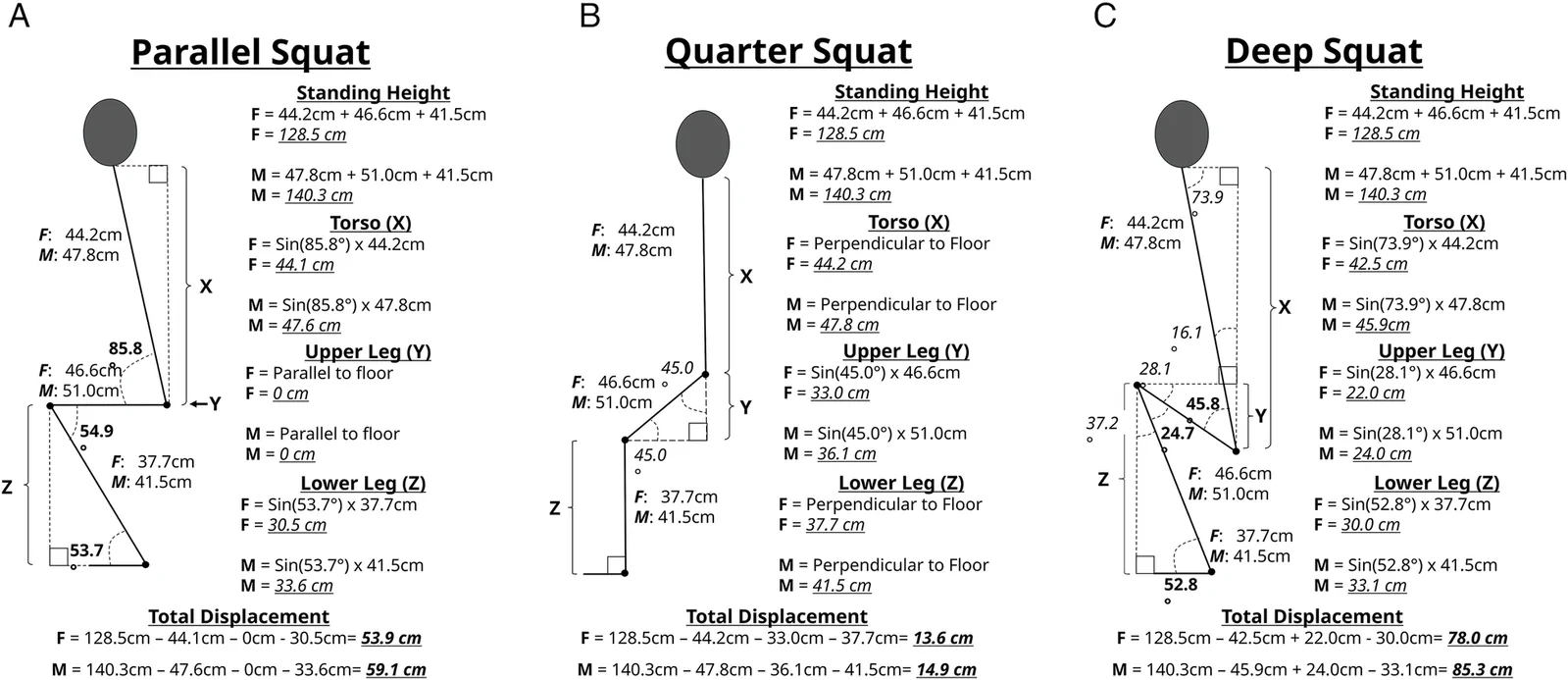
Determination of squat displacement. Squat displacement was determined for Parallel **(A)**, Quarter **(B)** and Deep **(C)** squats. Bolded angles indicate angles that were directly assessed through citation (Parallel Squats) or Kinematic Analysis (Deep Squat). Italicized angles indicate the angles were determined through standard triangle geometry. Standard typeface angles indicate angles that were assigned due to the nature of the exercise (Quarter Squat).

The percentage of body mass was set at 88% for all exercises involving the squat motion to represent all portions of the body contributing to changes in COM (i.e., everything except the shin, ankle, and foot) (47). Accordingly, the percentage of body mass for walking lunges was set at 84% to account for slightly less mass contributing to vertical changes in COM. Regarding mechanical work, the vertical distance of a lunge was estimated as the length of the shank. However, because repetitions are commonly awarded based on distance increments, repetitions cannot be counted like other exercises. Song et al. (51) reported that optimal step length of a lunge was roughly equal to the length of the lower-leg (51). Therefore, a reliable estimate for the number of lunges within an assigned space could be found by dividing leg length into distance travelled.

When calculating a WOD's cumulative workload, it is important to keep in mind that multiple variations of movement standards may be considered acceptable (3). Unless squat depth is specifically stated, power or squat cleans and snatches are allowed. Likewise, it does not matter whether a shoulder press, push press, push jerk, or split jerk is employed when the lift requires the external load to finish overhead with arms, hips, and legs extended. This flexibility means little in retrospect because the specific mechanical work calculation is based on observed technique. However, the exact workload cannot be known when workloads involving technical variation are prescribed in advance, or when quantifying completed workouts where technical variation might have occurred. In acknowledgement of that uncertainty, all calculations presented here assume the least complex version that would likely produce the fastest repetition completion rate for each exercise. For example, power cleans and snatches were assumed over squat cleans and snatches, and push press was assumed over push and split jerks. The same rationale was used when choosing to treat the shoulder-to-overhead exercise to be the same as push press and jerks, and the ground-to-overhead exercise the same as a power clean and jerk.

Finally, readers will note instances where several exercises were treated the same while other related exercises were not. For instance, though ground-to-overheads, clean and jerks, and snatches all begin at the floor and end with the arms, hips, and knees extended, the snatch was distinguished by grip width. A common method for finding an appropriate grip width for the snatch exercise is to find a position that allows the barbell to sit within the hip crease when the elbows are at full extension (52). This results in the final height of the barbell being lower during the snatch exercise compared to the other exercises. Thus, torso length was used to identify the height of the overhead barbell during the snatch, and the same criteria should be added to the first repetition of an overhead squat, unless observed otherwise (e.g., clean and jerk followed by spreading out the hands). In contrast, arm length determined the final position of ground-to-overheads and jerks. Meanwhile, jerks and push press were treated the same because vertical displacement of the barbell is ultimately the same, and changes in COM during a split jerk is closer to the quarter squat depth used for the push press than a parallel squat (53). Finally, front and overhead squat displacements are calculated the same despite their differences in difficulty. Although the overhead squat requires greater sustained activation of shoulder and scapular stabilizers (54) and the metabolic cost of isometric force maintenance is greater (55, 56), these do not change COM displacement after the barbell has been positioned overhead. Therefore, while single efforts should be calculated differently, the only differences in effort on subsequent repetitions have to do with metabolic costs.

### Plyometrics

The common HIFT exercises that would fall into the plyometric category are presented in Table 2. These types of exercises are traditionally defined as quick, powerful movements that utilize the stretch-shortening cycle via a rapid pre-stretch or countermovement (57). They are traditionally incorporated into training for the purpose of improving rate of force development and developing speed and power. However, these enhancements should not be considered expected outcomes in HIFT because they are not executed with recommended prescription. Whereas traditional prescription focuses on maximizing execution velocity by limiting fatigue via smaller repetition counts per set and overall, and then allotting sufficient rest intervals (57), HIFT prescription treats these more like conditioning. Prescribed repetition counts will often be much larger than traditional recommendations and the movements are placed within circuits that are not conducive for recovery (3). Though it is beyond the scope of this compendium to comment on safety, caution is advised when performing plyometric exercises, particularly box jumps, while fatigued.

**Table 2:** Mechanical work equations for common plyometric exercises.

Exercise	Standard Body Mass	Standardized Concentric Displacement Estimate			Mechanical work formula	Notes
		Measurement	Men	Women		
Box Jumps or Step-ups	100% Men = 85.6 kg Women = 62.6 kg	Box height + Quarter Squat Distance	0.759 m	0.644 m	(Body mass × Standard Distance) + (External load × Standard Distance)	Most common box height
						Men = 0.610 m
Box Jump-over or Step-over		Box height	0.610 m	0.508 m	(Body mass × Standard Distance) + (External load × Standard Distance)	Women = 0.508 m
Rope Jumping						
Single-unders		[2π (Wrist + Hand)]	0.390 m	0.340 m	(Wrist Rotation × Rope Mass) + (Jump Height × Body Mass)	Standard Jump Height = 0.064 m
Standard mass of jump rope = 0.1 kg						
Double Unders		[2π (Hand length)] × 2	0.780 m	0.679 m	(Wrist Rotation × Rope Mass) + (Jump Height × Body Mass)	
Wall Balls						
Pick-up	88% Men = 75.3 kg Women = 55.1 kg	Height—Plate Radius—Head—Neck—(0.5 × Wrist + Hand)	1.204 m	1.100 m	(Body Mass × Quarter Squat Distance) + (Medicine Ball Mass × Standard Distance)	
Shot		(Target Height—(Height—Head—Neck)) + Parallel Squat Distance	1.508 m	1.315 m	(Body Mass × Parallel Squat Distance) + (Medicine Ball Mass × Standard Distance)	Target Height: Men = 3.048 m; Women = 2.743 m

Although the plyometric category only consists of three basic exercises, several variations and combinations are allowed or prescribed (3). Table 2 presents some of these variations together, and it also distinguishes the mechanical work calculations for others. For instance, stepping and jumping onto a plyo box (standard dimensions = 0.762 m × 0.610 m × 0.508 m) require athletes to stand erect atop the box to complete each repetition while jump- and step-overs do not. The most common prescription heights for men (0.610 m) and women (0.508 m) were used for calculation purposes. Double-unders have always been the rope jumping variation prescribed in main division events, whereas traditional single-unders have been the primary scaling option (3). The main calculation differences between these variations are the number of wrist rotations and jump height (single-unders = 0.064 m; double-unders = 0.127 m). The jump rope (e.g., SR-343 Mach Speed Rope, Rogue Fitness, Columbus, OH, USA; ∼0.1 kg) is the same. Wall ball loads (men = 9.072 kg; women = 6.350 kg) and targets (men = 3.048 m; women = 2.743 m) have followed a similar pattern where main division prescription was altered slightly for scaled divisions (3), and the 0.18 m radius used for the pick-up calculation was based on a common brand (Rogue Fitness, Columbus, OH, USA).

### Gymnastics and calisthenics exercises

Exercises falling within this category may be divided into two main categories: those performed on the floor or against a wall, and those requiring a pull-up bar. The equations for the gymnastics and calisthenics exercises that often appear in the CFO are presented in Table 3.

**Table 3:** Mechanical work equations for common calisthenics and gymnastics exercises.

Exercise	Standard Body Mass	Standardized Concentric Displacement Estimate			Mechanical work formula	Notes
		Measurement	Men	Women		
Burpee						
Traditional	88% Men = 75.3 kg Women = 55.1 kg	Deep Squat + 0.0254 m	0.878 m	0.805 m	(Body Mass [72.1%] × Push-up Distance) + (Body Mass [88%] × Standard Distance)	
Bar-Facing (or Lateral)		Deep Squat + 0.2504 m	1.103 m	1.030 m		Barbell plate radius (0.225 m) + Clearance (0.0254 m)
Over Dumbbell		Deep Squat + 0.060 m	0.913 m	0.840 m		Dumbbell Diameter (0.035 m) + Clearance (0.0254 m)
Handstand						
Push-ups (Strict)	100% Men = 85.6 kg Women = 62.6 kg	Arm—Head + Neck	0.350 m	0.323 m	Body Mass × Standard Distance	
Kipping adjustment					(Body Mass × Standard Distance) × 0.72	
Walking and Wall Walks		0.085 × Arm	0.060 m	0.056 m	(Body Mass × Standard Distance) × (Distance ÷ (0.25 × Arm))	Standard Wall Walk Distance (Men = 3.048 m; Women = 2.794 m)
Muscle-ups						
Bar or Ring (Strict)	100% Men = 85.6 kg Women = 62.6 kg	(2 × Arm) + Wrist-hand	1.480 m	1.362 m	Body Mass × Standard Distance	
Kipping adjustment					(Body Mass × Standard Distance) × 0.84	
Pull-ups						
Traditional (Strict)	100% Men = 85.6 kg Women = 62.6 kg	Arm + (0.5 × [Wrist + Hand])—Head	0.504 m	0.463 m	Body Mass × Standard Distance	Standard head (men = 0.236 m; women = 0.218) and neck (men = 0.123 m; women = 0.113 m) lengths
Kipping adjustment					(Body Mass × Standard Distance) × 0.53	
Chest-to-bar (Strict)		Arm + (0.5 × [Wrist + Hand])	0.740 m	0.681 m	Body Mass × Standard Distance	
Kipping adjustment					(Body Mass × Standard Distance) × 0.68	
Push-ups	72.1% Men = 61.7 kg Women = 45.1 kg	Arm	0.709 m	0.654 m	Body Mass × Standard Distance	
Single-leg Squat	88% Men = 75.3 kg Women = 55.1 kg	Deep Squat Distance	0.853 m	0.780 m	Body Mass × Standard Distance	
Toes-to-bar						
Strict	33% Men = 27.9 kg Women = 20.4 kg	Leg radius × (1—(((Arm + Torso)—Leg)/Leg)	0.308 m	0.270 m	Body Mass × Standard Distance	
Kipping adjustment					(Body Mass × Standard Distance) × 0.21	

Push-ups are one of the most recognizable and foundational calisthenic exercises (58). They do not require equipment, have many variations, and are commonly employed as part of fitness tests in schools and tactical populations. Ignoring all horizontal displacement, mechanical work for the standard push-up is calculated as arm length (not counting wrist + hand) multiplied by 72.1% of body mass. Full arm length is used to account for scapular retraction and protraction in the down and up positions, while 72.1% is the average of reported body mass percentages in both positions (59). However, as a standalone exercise, the standard push-up has only been programmed once in Rx division of CFO competitions (i.e., only 11.2). Its mechanical work calculation is most relevant here because a push-up serves as the base movement for a burpee. Burpees have been programmed 14 times in 15 CFO competitions either as their own exercise or paired with other exercises such as box jumps, pull-ups, or jumping over a barbell or dumbbell (3). The concentric mechanical work calculation for the standard burpee progresses from a push-up to a deep squat and finishes with a jump. With no standard jump height for burpees, the calculations presented here are based on a 1-inch (0.0254 m) jump. This jump height represents the least amount of work required to meet movement standards. When burpees are paired with another exercise, the mechanical work calculation should either be adjusted for jump height (i.e., over a barbell or dumbbell, see Table 3) or added to the calculation for the paired movement (e.g., pull-up, box jump/jump-over).

The handstand push-up is the more commonly programmed variation of the push-up in CFO competition (3). It is performed from an inverted position while leaning against a wall or similar vertical surface. The concentric portion of the exercise begins with the crown of the athlete's head in contact with the floor and finishes with full arm extension. Though seemingly straightforward, different methods for determining full ROM have been enforced over the years (3), presumably to address various methods employed to reduce ROM and make the exercise easier. The method presented here bypasses more specific measurements and relies on the assumption that the athlete is in a near-vertical position and their hand-placement mirrors what they would use for a shoulder press, push press, or jerk (i.e., arm length minus head + neck length).

The handstand push-up calculations are also presented as a base calculation for performing the exercise strictly, along with an adjustment for kipping. The kip allowed for handstand push-ups should not be confused with that which is used for pull-ups and muscle-ups. Rather, the kip of a handstand push-up artificially lowers COM to provide space that enables momentum to be produced from a lower-body kick. That momentum occurs prior to when the upper-body musculature exerts concentric forces to raise COM against gravity. Although no study has compared strict to kipping handstand push-ups, the effect of the kip on mechanical work may be inferred from a recent study examining axial forces on the head and neck (60). Based on reported mean kipping peak force (991 N) and estimated force requirements for a strict handstand push-up based on mean body mass (79.0 kg × 9.81 m·sec^−2^ = 775 N), it is estimated that kipping reduces concentric force requirements by approximately 28%. Thus, we have assumed that mechanical work of kipping handstand push-ups is roughly equal to 72% of strict handstand push-ups.

The final variations from this line of exercise involve handstand wall walks and free handstand walking (3). Wall walks have athletes start lying face down on the floor with their feet against the wall and then progressing to an inverted, vertical position by walking their feet and hands up and towards the wall. Handstand walking is performed from an inverted position away from any vertical structures, and has the athlete travel a specified distance on their hands and while remaining in the inverted position. The most obvious difference, relevant to mechanical work, is the presence of the wall being used to support a percentage of the athlete's body weight. However, the supported body mass percentage will change with each hand step towards the wall, and it is thus, nearly impossible to generalize. Further, doing so would result in an overcomplicated equation that would defeat the purpose of this compendium (i.e., to provide simplified methods for calculating workload). Therefore, for simplicity, we have proposed that mechanical work per step be estimated the same way for both wall walks and handstand walking. When expressed in kgf·m, concentric mechanical work on each step is calculated as body mass multiplied by the vertical rise in COM. An inverted-pendulum model, commonly accepted in locomotion research (61, 62), is used to approximate the vertical change in COM to be approximately 5%–12% (mean = 8.5%) of arm length (63, 64). Then, total mechanical work performed on each official repetition (i.e., one wall walk, or the specified handstand walking distance) is found by multiplying the mechanical work performed on each step by the total number of steps. This is found by dividing the total required distance by the distance covered on each step. It is estimated that, depending on skill, the distance covered on each step ranges between 18% and 35% (∼25% of arm length is used here) (63, 65). This corresponds to the shorter, choppy steps commonly coached to help the athlete better maintain balance. Meanwhile, the total distance traveled is either specified by programming (for handstand walking) or is made standard (3). Since CFO movement standards have specific requirements about lowering the body during the eccentric phase of wall walks, the standard distances presented here (men = 3.048 m; women = 2.794 m) account for total distance travelled while raising and lowering the body.

The other common line of gymnastics/calisthenics exercises appearing in CFO programming are those performed on a pull-up bar. There is more variation in mechanical work and force expression during these movements because technical standards allow them to be performed in multiple ways (3). For instance, athletes are typically free to utilize a strict, kipping, or butterfly technique whenever traditional and chest-to-bar pull-ups are prescribed. Compared to strict technique, the kip and butterfly motions take advantage of momentum to reduce the force and energy needed to complete a single repetition (66). A kip creates a more rapid stretch between the eccentric and concentric phases, much like what occurs during plyometrics, and allows elastic energy to contribute to force production (21, 22, 57, 62, 67). Consequently, we determined the gravitational potential energy that is generated during the kip and subtracted this from the total work required for each respective movement. Although elastic energy is also harnessed during the butterfly motion, the degree of contribution depends on consistency and skill. Through personal observation, we have noted that COM moves through an oval pattern, and that momentum can be aided by a leg kick. However, the nature and usage of this leg kick can vary between individuals and repetitions, and there is no information available describing ideal technique. Thus, for the time being, our recommendations assume a similar adjustment between the kip and butterfly technique, and for workload monitoring purposes, that an athlete's preferred technique remains consistent.

To determine the potential energy of the kip, a kinematic analysis was completed (Figure 3A) on a screenshot taken from a video depicting the maximal anterior horizontal displacement of the kip during a kipping pull-up (68). Briefly, the length and vertical displacement of each body segment was determined first (Figures 3B–E) and the lengths of the torso, thigh and shank (standing posture) were made relative to the lengths of the torso, thigh and shank of the standard female and male (Figure 1; Figure 3A). This correction was then applied to determine their respective standard displacements (Table 3), and then the potential energy of each segment was calculated based on the percent contribution of each segment to the total body mass previously defined by Dempster and Gaughran (47). The sum of these was used to quantify potential energy, and this calculation assumes 100% conservation since it would otherwise be impossible to determine without actual testing.

**Figure 3:**
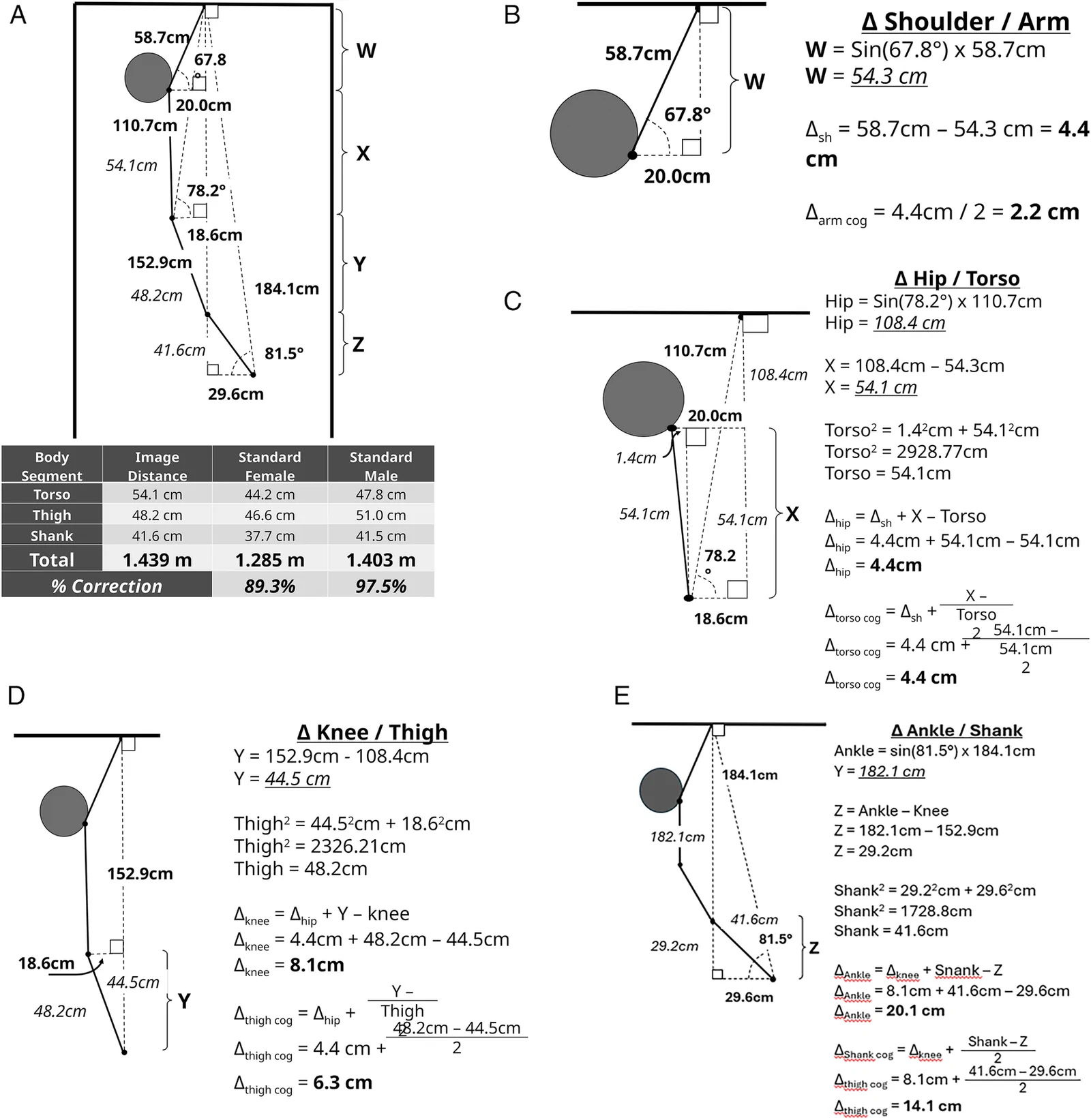
Determination of kip displacement. Displacement of each body segment during the kip was determined via kinematic analysis **(A)** All measured values are bold, while all calculated values are italicized. The change in the center of gravity (COG) for each body segment was calculated by the change in every superior body segment, as well as half of the change of that specific body segment. Each segment was broken down from the original analysis **(A)** Each component includes the change in the shoulder and arm COG **(B)**, hip and torso COG **(C)**, knee and thigh COG **(D)** and the ankle and shank COG **(E)** Once the vertical displacement of each segment was determined **(B–E)**, the measured length of each segment (torso, thigh and shank; standing posture) was made relative to the standard male and female lengths to apply a correction to each sex for body size **(A).**

Unlike many of the exercises described here, estimating mechanical work for toes-to-bar is tricky because it is not a simple linear motion. The exercise requires the athlete to begin from a hanging position and raise their toes to touch a pull-up bar, but the toes do not follow a straight path. There are three primary axes of rotation that lead the toes through an arced pathway. The first is where the hands grip the bar, and the degree of rotation occurring here is primarily affected by the usage and strength of the athlete's kip. If the athlete is utilizing a kip, this paper assumes the same contribution from the kip as described for pull-ups. The grip itself is an isometric effort that mainly contributes to the metabolic demand, and it is simpler to ignore this axis for the purposes of calculating mechanical work. The second and primar*y* axis is at the hips, and rotation at this joint is what allows the toes to travel in an arc up to the bar. The final axis is at the knee joint, and it only becomes involved in the final moment of repetition. Athletes who maintain a slightly flexed knee position, may need to kick their lower leg to allow their toes to contact the bar. Regardless, this would not change the vertical distance the toes must travel on each repetition. Consequently, only the lower-body mass (∼33% of total body mass) is relevant to the mechanical work calculation (47) and the focus is placed on hip rotation. Due to the impractical nature of quantifying angular rotation in a typical gym setting, the calculation presented here assumes that angles of rotation will not be known. Our approach begins by estimating COM of the lower-limb segments (as a percentage of distance from the proximal end) using adjusted Zatsiorsky-Seluyanov parameters for men (thigh = 40.95%, shin = 43.95%, ankle + heel = 44.15%) and women (thigh = 36.12%, shin = 43.52%, ankle + heel = 40.14%) (69). These values are entered into an equation that considers the mass and length of these segments to find leg radius (see Table 4 and Equation 1).

$$\mathrm{Leg}\;\mathrm{radius}=\;\frac{\left(A+B+C\right)}{D}$$ (1)

**Table 4:** Sub-component calculations for estimating leg radius for the toes-to-bar exercise.

Sub-component	Sex	Equation
A	Men	thigh mass (kg) × thigh length (m) × 0.4095
	Women	thigh mass (kg) × thigh length (m) × 0.3612
B	Men	shin mass (kg) × (thigh length (m) + (shin length (m) × 0.4395))
	Women	shin mass (kg) × (thigh length (m) + (shin length (m) × 0.4352))
C	Men	ankle-heel mass (kg) × (thigh length (m) + shin length (m) + (ankle-heel length (m) × 0.4415))
	Women	ankle-heel mass (kg) × (thigh length (m) + shin length (m) + (ankle-heel length (m) × 0.4014))
D	Men	thigh mass (kg) + shin mass (kg) + ankle-heel mass (kg)
	Women	

Then, leg radius is multiplied by the ratio between hip position at the end of concentric motion (quantified as the sum of the arm and torso) and leg length to find standard displacement (see Table 3). This approach mirrors established biomechanical modeling principles in gymnastics. Multi-segment models have been used to demonstrate how anthropometric proportions influence torque, COM changes, and dynamic motion (70). Likewise, inter-segmental coordination studies (71) and kinematic analyses of bar skills (72) show that ratios among limb and torso lengths significantly affect angular trajectories and effective lever arms.

#### Monostructural exercises

It is difficult to represent workload solely from a mechanical perspective for exercises falling within this category. Regardless of the specific modality (rowing, skiing, cycling, running), these exercises require continuous low-complexity, cyclical muscle actions (6). Sustained effort becomes increasingly difficult as the relative mechanical demand increases due to increased energy demands (24). With energy availability being significantly more important than force production, the specific amount of force exerted on each running stride or rowing stroke is not a typical consideration when programming these modalities. Instead, relative intensity is often associated with physiological (e.g., percentage of aerobic capacity or lactate threshold) and/or practical (i.e., percentage of racing pace) and the volume of work is simply described by distance or duration (6, 36). Of these, pace (i.e., distance over duration) is useful to workload calculations (35), but it is an unknown prior to exercise. Individual athletes can estimate their expected pace or completion time based on their personal experiences. However, estimating these for larger, heterogeneous groups becomes increasingly less reliable. Fortunately, these modalities are well-studied, and different approaches might be applied to estimate workload from given workout prescription.

#### Rowing, skiing, and cycling

All monostructural exercises are performed on an ergometer except for running (3, 73). Of those performed on an ergometer, rowing has been the most common modality to appear in competition followed by cycling and skiing at advanced stages. Performance in each is evaluated by either calories or meters completed. By themselves, calories and meters simply represent “repetitions” and are not sufficient for describing workload. However, post-exercise workloads can be derived from the information provided by the ergometer's performance monitor. Official competitions have limited rowing and skiing to a specific ergometer brand (Concept2, Morrisville, VE, USA), whereas three common ergometers have appeared in competition for cycling, the Echo Bike (Rogue Fitness, Columbus, OH, USA), the BikeErg (Concept2, Morrisville, VE, USA), and the Assault Bike (Assault Fitness, San Clemente, CA, USA). The sophistication of each ergometer's performance monitor and their ability to connect with supporting software varies across brands. Of all the brands, only Concept2 has publicly provided the basis for the estimates reported on their performance monitors (74). The other brands can be assumed to have done the same thing, but their equations are proprietary and not interchangeable with other brands. Thus, these data should be evaluated within, not between, brands. That is, fair comparisons can be made between different workloads performed on the same brand (e.g., Concept2 only, Rogue only, Assault Fitness only), but not between brands (e.g., between Concept2 and Rogue). To estimate workload (in kgf·m) from data reported on the performance monitor, equation 2 uses the product of average watts and duration (in seconds) to calculate energy (in joules) and then converts it to mechanical work (i.e., 1 joule = 0.102 kgf·m) (75).

Mechanicalwork(kgf⋅m)=AverageWatts×Duration(inseconds)×0.102 (2)

Advance estimates of workload require additional assumptions based on the principles of physics and cellular metabolism. When calories are known, they must first be corrected for body mass. Concept2 bases its estimate of calories off a 79.5 kg reference user (74), and this can be corrected for the exact body mass of individual athletes or average body mass of multiple athletes. Then, “true” calories can be expressed as energy (one calorie is equal to 4,184 joules) (75), and then a portion of energy (∼75%–80%) can be assumed to have been lost as heat if the individual or group consists of healthy adults (76, 77). The resultant value can then be converted to workload (i.e., 1 joule = 0.102 kgf·m) (75).

$$\mathrm{Mechanical}\;\mathrm{work}\;(\mathrm{kgf}\cdot m)=\left[\mathrm{Display}\;\mathrm{calories}\times \frac{\mathrm{Body}\;\mathrm{Mass}\;(\mathrm{kg})}{79.5}\right]\times 4184\times 0.225\times 0.102$$ (3)

It is important to note that this calculation will produce a lower workload estimate per calorie for smaller athletes. This is correct because larger athletes can theoretically provide more force per pedal revolution or stroke and spread the mechanical load across more body mass. These efforts feel more sustainable because each stroke or flywheel revolution leads to more distance being quantified and a lower metabolic cost.

When a specific distance (in m) is the known prescription variable (e.g., 500 m of rowing per round), completion time must be estimated or known to calculate work. The rowing ergometer links distance (in m) and calories through pace (74). The ergometer's microcomputer calculates power (watts) from 500-meter split time and converts it to calories per hour, and calories per hour can be divided by meters per hour to give calories per meter. Practically, this means Equation 3 can still be used if assigned meters are converted to calories using estimated 500 m split times. For example, 1 meter at a 2:30 split equals ∼0.055 calories, ∼0.066 calories at a 2:00 split, and ∼0.078 calories at a 1:45 split. Estimated total calories from assigned meters (Equation 4) and expected average 500 m split time can then be inserted into Equation 3 to calculate total work. Average 500 m split times during a 2,000 m rowing test for recreational HIFT men (1:52, 0.072 calories per meter) and women (2:08, 0.062 calories per meter) (78) were used for calculations performed in this paper.

$$\mathrm{Calories}\;\mathrm{per}\;\mathrm{meter}=\;\frac{300+\frac{4}{1.1639}\times \frac{2.8}{{\left(\frac{500m\;\mathrm{split}[\mathrm{seconds}]}{500}\right)}^{3}}}{\frac{500\times 3600}{500m\;\mathrm{split}\;[\mathrm{seconds}]}}$$ (4)

Estimated pace combined with an assumption about the ergometer's calibration can also be used to calculate total work, but this alternative would not factor athlete body mass. Concept2 uses a known constant (2.8 kg) based on empirical calibration that considers air resistance, flywheel mechanics, and the ergometer's drag factor (79). Equation 5 uses that constant, an energy to workload conversion factor (i.e., 1 joule = 0.102 kgf·m) (75), and an estimated completion time (e.g., from an average 500-m split time), to calculate mechanical work (in kgf·m).

$$\mathrm{Mechanical}\;\mathrm{work}\;(\mathrm{kgf}\cdot m)=0.2856\times \frac{\mathrm{Distance}\;{\left(m\right)}^{3}}{\mathrm{Duration}\;{\left(\mathrm{seconds}\right)}^{2}\;}$$ (5)

#### Running

When running has been programmed into a WOD, performance is based on distance and scored as a single repetition per distance increment (e.g., one repetition every 50 feet [15.24 m]) (3, 73). The mechanical work performed when running a given distance can be estimated using a known approximation (0.25 kilocalories of external mechanical work per kilogram per kilometer) (80), which represents the energy required to lift and accelerate COM during level-ground running. Equation 6 accounts for individual variations in efficiency (∼0.45 kcal·kg^−1^·km^−1^) due to body mass (80) and estimates mechanical work (in kgf·m) by converting kilocalories (1 kilocalorie = 426.83 kgf·m) (75).

Mechanicalwork(kgf⋅m)=Bodymass(kg)×[0.001×distance(m)]×192.1 (6)

### Examples

Tables 5, 6 utilize the standard equations described here to provide estimates of mechanical work and work rate for select CrossFit® Open workouts and the mechanical work contributions of their respective individual exercises. The workouts were selected from those prescribed in the CrossFit® Open, and work calculations are based on a 50th percentile score (16, 17). Assigned programming was used to decompose the 50th percentile score and determine the exact number of repetitions completed for each exercise. Repetitions for each exercise were then multiplied by the estimated work performed during a single repetition. The estimated work was based on the estimated vertical travel distance of the loads (internal and external) lifted by the average male and female athlete (15–17, 41). The estimated positive external mechanical work for each exercise was expressed in a common unit (kgf·m) and summed to estimate total workout work. Total work was then divided by workout duration to calculate work rate (kgf·m·min^−1^). Although different models were used across exercise types, expressing each estimate in the same unit allowed them to be combined for the overall workload calculation. Doing so does not imply that the same amount of mechanical work performed on different exercise modalities produces the same metabolic, neuromuscular, or tissue demand. Rather, total estimated work should be interpreted alongside the individual contributions of each exercise (see Table 6).

**Table 5:** Example total mechanical work and work rate calculations for select crossFit® open workouts using 50th percentile scores for men and women.

Workout (score)	Prescription (men/women)	Repetitions		Time (min)		Work (kgf·m)		Work Rate (kgf·m·min^−1^)	
		Men	Women	Men	Women	Men	Women	Men	Women
13.4 (repetitions)	7 min AMRAP, *Add 3 repetitions after each completed set								
	3 × Clean and jerk (135lbs/95lbs)	70	64	7		5,900	3,244	843	463
	3 × Toes-to-bar								
15.2 (repetitions)	3 min to complete 2 sets, *Add another 3 min and 2 repetitions after completing 2 sets within the time limit								
	10 × Overhead squats (95lbs/65lbs)	127	133	9		7,724	5,483	858	609
	10 × Chest-to-bar pull-ups								
17.4 (repetitions)	13 min AMRAP								
	55 × Deadlifts (225lbs/155lbs)	177	173	13		11,742	7,963	903	613
	55 × Wall ball shots (20lbs/14lbs to 10’/9’ target)								
	55-calorie Rowing								
	55 × Handstand push-ups								
19.4 (TTC → repetitions)	12 min time limit to complete 3 sets of:								
	10 × Snatches (95lbs/65lbs)	93	67	12 (9 min of exercise)		10,437	4,658	870 (1,160)	388 (518)
	12 × Bar-facing burpees								
	Then, rest 3 min before completing 3 sets of:								
	10 × Bar muscle-ups								
	12 × Bar-facing burpees								
21.2 (TTC → repetitions)	20 min time limit, alternate the following:								
	Dumbbell snatches (50lbs/35lbs): ×10 → 20 → 30 → 40 → 50 repetitions	225		15.98	18.58	20,730	14,303	1,297	770
	15 × Burpee box jump-overs (“24/20”)								
23.2a (time)	15 min AMRAP, *Add 5 burpees after each round								
	5 × Burpee pull-ups	125	112	15		24,005	15,516	1,600	1,034
	10 × 50-ft shuttle run								
23.2b (load)	Then, 5 min to find:	196	129	5		274	169	55	34
	1-RM Thruster								

**Table 6:** Contribution of individual exercises to total mechanical work in select crossFit® open workouts.

Workout (score)	Prescription (men/women)	Men				Women			
		Mechanical Work (kgf·m)				Mechanical Work (kgf·m)			
		Repetitions	from Floor	Exercise	Total	Repetitions	from Floor	Exercise	Total
13.4 (repetitions)	7 min AMRAP, *Add 3 repetitions after each completed set								
	3 × Clean and jerk (135lbs/95lbs)	40			5,833	34			3,209
	3 × Toes-to-bar	30			67	30			35
15.2 (repetitions)	3 min to complete 2 sets, *Add another 3 min and 2 repetitions after completing 2 sets within the time limit								
	10 × Overhead squats (95lbs/65lbs)	69	4,828	397	5,226	72	3,229	486	3,715
	10 × Chest-to-bar pull-ups	58			2,498	61			1,768
17.4 (repetitions)	13 min AMRAP								
	55 × Deadlifts (225lbs/155lbs)	55			2,572	55			1,578
	55 × Wall ball shots (20lbs/14lbs to 10’/9’ target)	55	3,202	22	3,225	55	2,096	14	2,111
	55-calorie Rowing	55			5,686	55			4,159
	55 × Handstand push-ups	12			259	8			116
19.4 (TTC → repetitions)	12 min time limit to complete 3 sets of:								
	10 × Snatches (95lbs/65lbs)	30			2,754	30			1,482
	12 × Bar-facing burpees	36			4,565	36			3,105
	Then, rest 3 min before completing 3 sets of:								
	10 × Bar muscle-ups	15			1,596	1			72
	12 × Bar-facing burpees	12			1,522	0			
21.2 (TTC → repetitions)	20 min time limit, alternate the following:								
	Dumbbell snatches (50lbs/35lbs): ×10 → 20 → 30 → 40 → 50 repetitions	150			8,575	150			5,565
	15 × Burpee box jump-overs (“24/20”)	75			12,156	75			8,738
23.2a (time)	15 min AMRAP, *Add 5 burpees after each round								
	5 × Burpee pull-ups	75			11,475	72			8,186
	10 × 50-ft shuttle run	50 (762 m)			12,530	40 (610 m)			7,331
23.2b (load)	Then, 5 min to find:								
	1-RM Thruster	114	114	160	274	69	69	100	169

For comparisons among athletes of different body sizes, mechanical work and work rate may also be expressed relative to body mass. They are respectively calculated by dividing mechanical work and work rate values by body mass (i.e., kgf·m·kg^−1^ and kgf·m·kg^−1^·min^−1^). Calculating these normalized indices reduces the direct influence of body mass. However, they do not account for variability arising from differences in body proportions, technique, skill, or externally prescribed loads. Accordingly, normalized values should complement rather than replace absolute estimates, and they should not be interpreted as measures of relative physiological intensity. Body-mass-normalized mechanical work and work rate for the example CrossFit® Open workouts and exercise-specific contributions presented in Tables 5, 6 can be found in Supplementary Table S1.

### Anthropometric sensitivity analysis

Anthropometric variation affected estimated mechanical work differently across exercises (Supplementary Table S2). Changing stature alone had no effect on rowing, changed box jump estimates by less than 1%, and changed thruster, burpee, and kipping pull-up estimates by approximately 4%. Changing body mass alone altered thruster estimates by approximately 4% and the other exercises by approximately 9%. The largest differences occurred when stature and body mass were changed together, with estimates ranging from approximately 13% below to 14% above the standard models for burpees and kipping pull-ups. Because stature is not included in the rowing equation, combined changes were identical to those produced by body mass alone (approximately ±9%).

## Limitations and practical applications

This paper provides instructions on how to estimate mechanical work for exercises that commonly appear in HIFT programming. However, there are several limitations and assumptions that must be acknowledged.

1. This compendium addresses about fifty individual exercises or their variations but is primarily concerned with those appearing in the CrossFit® Open (3). These were selected because their respective competition-enforced movement standards are known and can be expected to be employed by athletes during normal training. However, many other exercises, known to appear in training and competition (e.g., kettlebell swings, glute ham developer [GHD] sit-ups, rope and peg board ascents) were excluded because of a lack of published standards or a more drastic impact from technical variations. For example, the vertical distance travelled during a kettlebell swing is affected by style (i.e., “American” vs. “Russian”), and successful execution of movement standards can be impacted by subjective perception of height and compensatory motion. Similarly, the settings of the GHD machine are not made standard and these too could impact vertical distance travelled in GHD sit-ups. Meanwhile, though the mechanical work of rope and peg board ascents may be found by multiplying body mass and rope height, the starting point of the initial pull (i.e., initial arm extension with or without a jump) and technical variations in subsequent pulls make it difficult to precisely identify which body regions are performing that work. Consequently, this compendium will require periodic updates as these movements and others become better understood.
2. Mechanical work calculations assume that repetitions are performed exactly as described by published movement standards (3) and with optimal efficiency (i.e., no wasted motion). For example, power cleans and snatches assume a quarter squat, whereas a parallel squat (not a deep squat) was considered when calculating standard distances for squat cleans and snatches. These squat depths represent the minimum vertical distance travel requirement to meet movement standards, assume biomechanically efficient trunk flexion, and do not consider the impact of base variation (i.e., stance width on distance requirements). These standardized models should therefore be viewed as minimum-displacement estimates rather than exact representations of each athlete's movement. Differences in technique may cause estimated mechanical work to be either higher or lower than the actual value, and these differences should not be assumed to cancel across repetitions. Uncertainty will generally be greatest when technical variation affects the primary component of the exercise-specific calculation: external-load displacement during loaded exercises; body mass and center-of-mass displacement during gymnastics, calisthenic, and plyometric exercises; and pace, calibration, or device-specific assumptions during monostructural movements. Consistent deviations from the standardized model, such as deeper squats, greater jump heights, reduced range of motion, or different kipping strategies, may therefore produce systematic differences between estimated and actual work. When athlete-specific displacement or technique can be directly measured, those values should be used in place of the standardized inputs.

Practical correction of technical variation. When a repetition meets movement standards but displacement differs from the standardized estimate and can be measured, the observed value should replace the standardized value in the equation. This can also be expressed as: corrected work = standardized work × (observed displacement ÷ standardized displacement). For exercises with separate body-mass and external-load displacement terms, each should be corrected separately unless both change proportionally. Note that replacing standardized displacement with an observed value does not negate the importance of selecting the most appropriate model. For instance, it would not be appropriate to apply a correction coefficient to the squat clean model if the athlete instead performs a power clean or to the strict pull-up model if the athlete kips. When technique changes enough to meet the criteria of another exercise, the model for that exercise should be used. That said, when technical variation is evident but cannot be quantified, the standardized value may still be reported but should not be considered an individualized estimate. Future studies using video or wearable sensors may allow exercise-specific correction coefficients to be developed.

3. All standard distances were based on body dimensions estimated from average self-reported body mass and stature values for male and female competitors (81). Because these values were self-reported, their accuracy and timeliness could not be confirmed in the studies that later used them (15–17, 41), which introduces error in body proportion estimates and standard distance calculations. That said, since these proportions were consistently applied in all equations presented here. Using them to estimate expected mechanical work to make purposeful adjustments to the programming is still appropriate. Furthermore, each equation indicates the relevant body regions that would need to be measured (length in m, mass in kg) to find more precise estimates of mechanical work. Accordingly, the standard male and female anthropometric models are most appropriate for general programming or workout-level comparisons when individual measurements are not available. When addressing the needs of specific athletes, body mass and the regional lengths identified in Tables 1–4 should be measured whenever possible and used in place of the standard value. Regional lengths can still be estimated using the same proportional procedures used to develop the standard male and female models if only stature is known. Because anthropometric characteristics and habitual movement technique are likely to be more consistent within an athlete than between athletes, repeated within-athlete comparisons may be more meaningful than direct between-athlete comparisons based only on standardized anthropometric models.
4. Body mass and/or external load were the main determinants of force expression, and these values do not account for acceleration or pre-existing momentum (21, 23, 82). Rapid acceleration of a load, via intention, elastic energy, and/or reaction forces, generates momentum that can alter the force required for successful execution (67). While these strategies mainly reduce time under tension and are most relevant to metabolic work requirements (55, 56, 76, 77), they also reduce the portion of motion where the musculature must work to accelerate the load against gravity. Knowing the exact effect such motions have on mechanical work would require kinetics testing at different points of a movement (via motion capture or force plate). In lieu of that information, and to maintain consistency with other assumptions, the “kipping” adjustments presented here assume a 100% conservation of energy.
5. The calculations presented here are limited to positive external mechanical work and, for discrete vertical exercises, primarily reflect the concentric phase. Negative external work during the eccentric phase was not estimated because published movement standards typically define repetition endpoints but do not standardize descent velocity, movement trajectory, braking strategy, rebound, impact, or the extent to which an external load is actively controlled during the descent. Applying a single eccentric-work correction across HIFT exercises would therefore require additional assumptions that are not currently supported. As a result, these values should not be interpreted as estimates of total absolute mechanical work, eccentric muscular work, or eccentric musculoskeletal loading. Future studies using force platforms and motion capture, potentially supplemented by wearable sensors, will be needed to quantify negative work and determine whether exercise-specific eccentric corrections are appropriate.
6. Work calculations also ignore the contribution of horizontal resistance. These are primarily relevant to and represent the primary resistive force when pushing or pulling an object (i.e., a sled) along the ground. However, although such examples may be found in advanced stages of the CrossFit® Games competition (<4% of events have programmed a sled) (3), their appearance during normal training sessions is unknown but would seem less common due to logistics (e.g., sled availability for all class attendees). Their exclusion here is mainly due to the impact of environment and sled type variation on work calculations limiting any ability to offer generalized estimates.
7. Metabolic work and physiological stress are important components of the overall demands of a workout (20–23), but they were not directly measured or estimated for the present framework. Mechanical work rate describes the rate at which the estimated positive external mechanical work is completed and should not be interpreted as a direct measure of fatigue, metabolic stress, relative intensity, or tissue loading. Associations between mechanical work or work rate and physiological responses are likely to depend on individual physiological characteristics (e.g., aerobic capacity, lactate threshold, anaerobic capacity, maximal strength), technical skill, exercise modality, and workout structure. Future research may determine whether expressing work rate relative to an athlete's maximal exercise- or round-completion speed improves its relationship with physiological responses (35), though this remains a hypothesis requiring empirical validation.

Quantifying work and work rate requirements in HIFT is not a new concept but it is not commonly or easily practiced. HIFT workouts might consist of any combination of exercises drawn from a wide variety of modalities. The biomechanical methods used to estimate work differ across modalities and sufficient understanding of these concepts is not a prerequisite to being a successful HIFT coach or athlete. Since every workout is likely to require a different calculation, it is understandable that coaches might avoid this practice because they view it as being overly tedious and unnecessary. However, doing so limits their ability to make objective and purposeful adjustments to programming. The equations presented in this compendium provide coaches and athletes with simplified instructions for estimating the mechanical work and work rate required of most HIFT workouts. Although these equations do not account for exercise complexity or metabolic stress, they still allow work requirements to be compared across workout designs. Coaches and athletes can use this information to more clearly identify work requirements in competition, and to monitor completed training workloads and adjust prescriptions within and across training cycles. Furthermore, researchers can utilize these instructions to more clearly report the estimated external mechanical demands imposed by study interventions and compare those demands across studies using different HIFT-style workouts. Classifying workouts according to estimated mechanical work and work rate may also provide a standardized framework for future research examining whether physiological responses are consistent among workouts with similar mechanical demands.
