# Supplementary material for: Standard methods for estimating mechanical work in high-intensity functional training: an exercise compendium
Source: Front Sports Act Living. 2026 Oct 27;8:1974999. doi: 10.3389/fspor.2026.1974999 (PMC13749379; doi:10.3389/fspor.2026.1974999)
Supplement: Supplementary file 1 [file Table1.docx]

**Supplementary Table S1. Body-mass-normalized mechanical work and work rate for the example CrossFit® Open workouts and exercise-specific contributions presented in Tables 5 and 6.**

|  |  | **Men** | | |  | **Women** | | |
| --- | --- | --- | --- | --- | --- | --- | --- | --- |
|  |  | **Mechanical Work (kgf·m·kg^-1^)** | | **Work Rate** |  | **Mechanical Work (kgf·m·kg^-1^)** | | **Work Rate** |
| **Workout (score)** | **Prescription (men / women)** | **Exercise** | **Workout** | **(kgf·m·kg^-1^·min^-1^)** |  | **Exercise** | **Workout** | **(kgf·m·kg^-1^·min^-1^)** |
| 13.4 (repetitions) | *7-minute AMRAP, *Add 3 repetitions after each completed set* | | | | | |  |  |
|  | 3 x Clean and jerk (135lbs / 95lbs) | 68.1 | 68.9 | 9.8 |  | 37.5 | 51.8 | 7.4 |
|  | 3 x Toes-to-bar | 0.8 |  |  |  | 0.4 |  |  |
| 15.2 (repetitions) | *3-minutes to complete 2 sets, *Add another 3 minutes and 2 repetitions after completing 2 sets within the time limit* | | | | | |  |  |
|  | 10 x Overhead squats (95lbs / 65lbs) | 61.1 | 90.2 | 10.0 |  | 43.4 | 87.6 | 9.7 |
|  | 10 x Chest-to-bar pull-ups | 29.2 |  |  |  | 20.7 |  |  |
| 17.4 (repetitions) | *13-minute AMRAP* |  |  |  |  |  |  |  |
|  | 55 x Deadlifts (225lbs / 155lbs) | 30.0 | 137.2 | 10.5 |  | 18.4 | 127.2 | 9.8 |
|  | 55 x Wall ball shots (20lbs / 14lbs to 10' / 9' target) | 37.7 |  |  |  | 24.7 |  |  |
|  | 55-calorie Rowing | 66.4 |  |  |  | 48.6 |  |  |
|  | 55 x Handstand push-ups | 3.0 |  |  |  | 1.4 |  |  |
| 19.4 (TTC → repetitions) | *12-minute time limit to complete 3 sets of:* | | | | | |  |  |
|  | 10 x Snatches (95lbs / 65lbs) | 32.2 | 121.9 | 10.2 |  | 17.3 | 74.4 (13.6) | 6.2 (8.3) |
|  | 12 x Bar-facing burpees | 53.3 |  |  |  | 36.3 |  |  |
|  | *Then, rest 3 minutes before completing 3 sets of:* |  |  |  |  |  |  |  |
|  | 10 x Bar muscle-ups | 18.6 |  |  |  | 0.8 |  |  |
|  | 12 x Bar-facing burpees | 17.8 |  |  |  |  |  |  |
| 21.2 (TTC → repetitions) | *20-minute time limit, alternate the following:* | | | | | |  |  |
|  | Dumbbell snatches (50lbs / 35lbs): x 10 → 20 → 30 → 40 → 50 repetitions | 100.2 | 242.2 | 15.2 |  | 65.0 | 228.5 | 12.3 |
|  | 15 x Burpee box jump-overs (24" / 20") | 142.0 |  |  |  | 102.1 |  |  |
| 23.2a (time) | *15-minute AMRAP, *Add 5 burpees after each round* | | | | | |  |  |
|  | 5 x Burpee pull-ups | 134.1 | 280.4 | 18.7 |  | 95.6 | 247.9 | 16.5 |
|  | 10 x 50-ft shuttle run | 146.4 |  |  |  | 85.6 |  |  |
| 23.2b (load) | Then, 5 minutes to find: |  |  |  |  |  |  |  |
|  | 1-RM Thruster | 3.2 | 3.2 | 0.6 |  | 2.0 | 2.7 | 0.5 |

**Note. All work and work rate normalized to the body mass of the standard male (85.6 kg) and female (62.6 kg)**
