# Supplementary material for: Standard methods for estimating mechanical work in high-intensity functional training: an exercise compendium
Source: Front Sports Act Living. 2026 Oct 27;8:1974999. doi: 10.3389/fspor.2026.1974999 (PMC13749379; doi:10.3389/fspor.2026.1974999)
Supplement: Supplementary file 2 [file Table2.docx]

**Supplementary Table S2. Sensitivity of estimated mechanical work to ±1 SD variation in stature and body mass for representative high-intensity functional training exercises.**

|  | Standard | |  | Height (-1 SD) | |  | Height (+1 SD) | |  | Body mass (-1 SD) | |  | Body mass (+1 SD) | |  | Both (-1 SD) | |  | Both (+1 SD) | |
| --- | --- | --- | --- | --- | --- | --- | --- | --- | --- | --- | --- | --- | --- | --- | --- | --- | --- | --- | --- | --- |
|  | Men | Women |  | Men | Women |  | Men | Women |  | Men | Women |  | Men | Women |  | Men | Women |  | Men | Women |
| Height (cm) | 177.2 | 163.6 |  | 170.7 | 157.0 |  | 183.7 | 170.2 |  | 177.2 | 163.6 |  | 177.2 | 163.6 |  | 170.7 | 157.0 |  | 183.7 | 170.2 |
| Body mass (kg) | 85.6 | 62.6 |  | 85.6 | 62.6 |  | 85.6 | 62.6 |  | 77.7 | 56.8 |  | 93.5 | 68.4 |  | 77.7 | 56.8 |  | 93.5 | 68.4 |
| Barbell thrusters |  |  |  |  |  |  |  |  |  |  |  |  |  |  |  |  |  |  |  |  |
| Work (kgf·m) | 101 | 65 |  | 97 | 62 |  | 104 | 68 |  | 96 | 62 |  | 105 | 68 |  | 93 | 60 |  | 108 | 70 |
| Δ Standard (%) | 0.00 | 0.00 |  | -3.67 | -4.03 |  | 3.67 | 4.03 |  | -4.09 | -4.24 |  | 4.09 | 4.24 |  | -7.60 | -8.10 |  | 7.90 | 8.45 |
| Box jumps |  |  |  |  |  |  |  |  |  |  |  |  |  |  |  |  |  |  |  |  |
| Work (kgf·m) | 65 | 40 |  | 65 | 40 |  | 65 | 41 |  | 59 | 37 |  | 71 | 44 |  | 59 | 36 |  | 71 | 44 |
| Δ Standard (%) | 0.00 | 0.00 |  | -0.72 | -0.85 |  | 0.72 | 0.85 |  | -9.23 | -9.27 |  | 9.23 | 9.27 |  | -9.88 | -10.04 |  | 10.02 | 10.20 |
| Burpees |  |  |  |  |  |  |  |  |  |  |  |  |  |  |  |  |  |  |  |  |
| Work (kgf·m) | 110 | 74 |  | 106 | 71 |  | 114 | 77 |  | 100 | 67 |  | 120 | 81 |  | 96 | 64 |  | 124 | 84 |
| Δ Standard (%) | 0.00 | 0.00 |  | -3.60 | -4.00 |  | 3.60 | 4.00 |  | -9.20 | -9.30 |  | 9.20 | 9.30 |  | -12.50 | -12.86 |  | 13.17 | 13.59 |
| Kipping pull-ups |  |  |  |  |  |  |  |  |  |  |  |  |  |  |  |  |  |  |  |  |
| Work (kgf·m) | 23 | 15 |  | 22 | 15 |  | 24 | 16 |  | 21 | 14 |  | 25 | 17 |  | 20 | 13 |  | 26 | 17 |
| Δ Standard (%) | 0.00 | 0.00 |  | -3.67 | -4.03 |  | 3.67 | 4.03 |  | -9.23 | -9.27 |  | 9.23 | 9.27 |  | -12.56 | -12.93 |  | 13.24 | 13.67 |
| Rowing |  |  |  |  |  |  |  |  |  |  |  |  |  |  |  |  |  |  |  |  |
| Work (kgf·m) | 5686 | 4159 |  | 5686 | 4159 |  | 5686 | 4159 |  | 5162 | 3773 |  | 6211 | 4544 |  | 5162 | 3773 |  | 6211 | 4544 |
| Δ Standard (%) | 0.00 | 0.00 |  | 0.00 | 0.00 |  | 0.00 | 0.00 |  | -9.23 | -9.27 |  | 9.23 | 9.27 |  | -9.23 | -9.27 |  | 9.23 | 9.27 |

**Note. Standard anthropometric values were 177.2 cm and 85.6 kg for men and 163.6 cm and 62.6 kg for women. Sensitivity conditions independently and simultaneously varied stature or body mass by ±1 SD, and proportionally scaled body mass and length segments and displacements using the same methods described for the standard models. All other exercise-specific inputs were held constant. Calculations were based on 95-lb (43.1-kg) and 65-lb (29.5-kg) barbell thrusters, 24-in (0.610-m) and 20-in (0.508-m) box jumps, traditional burpees, traditional kipping pull-ups, and 55-calorie rowing for men and women, respectively. Δ Standard (%) represents the percentage difference from the corresponding standard male or female estimate. Because exercise duration was unchanged across sensitivity conditions, proportional changes in work rate are identical to those reported for mechanical work. SD = standard deviation.**
